# State Laws Requiring Hand Sanitation Stations at Animal Contact Exhibits—United States, March–April 2016

**DOI:** 10.15585/mmwr.mm6601a4

**Published:** 2017-01-13

**Authors:** Aila Hoss, Colin Basler, Lauren Stevenson, Kelly Gambino-Shirley, Misha Park Robyn, Megin Nichols

**Affiliations:** ^1^Public Health Law Program, Office for State, Tribal, Local and Territorial Support, CDC; ^2^Outbreak Response and Prevention Branch, Division of Foodborne, Waterborne, and Environmental Diseases, National Center for Emerging and Zoonotic Infectious Diseases, CDC; ^3^Oak Ridge Institute for Science and Education, Oak Ridge, Tennessee; ^4^Epidemic Intelligence Service, CDC; ^5^Preventive Medicine Residency and Fellowship, CDC.

In the United States, animal contact exhibits, such as petting zoos and agricultural fairs, have been sources of zoonotic infections, including infections with *Escherichia coli*, *Salmonella*, and *Cryptosporidium* (*1*–*4*). The National Association of State Public Health Veterinarians recommends handwashing after contact with animals as an effective prevention measure to disease transmission at these exhibits (*4*). This report provides a list of states that have used law, specifically statutes and regulations, as public health interventions to increase hand sanitation at animal contact exhibits. The report is based on an assessment conducted by CDC's Public Health Law Program, in collaboration with the Division of Foodborne, Waterborne, and Environmental Diseases in CDC’s National Center for Emerging and Zoonotic Infectious Diseases. The assessment found that seven states have used statutes or regulations to require hand sanitation stations at these exhibits (*5*). Jurisdictions seeking to improve rates of hand sanitation at animal contact exhibits can use this report as a resource in developing their own legal interventions.

A list of statutes and regulations was compiled using WestlawNext, an online legal research database, from March 17 to April 1, 2016. Before searching the database, literature on animal contact exhibits was examined to identify potential search terms. Search strings were created to capture the various terms used by states to refer to animal contact exhibits in their law. Only animal contact exhibit laws that specifically referenced hand sanitation were included in the assessment. The search was conducted in all 50 states and the District of Columbia and documented in a detailed research procedure. Relevant laws were then analyzed and coded. On June 2016, the findings of the assessment were emailed to public health veterinarians in 50 states and the District of Columbia. They were asked to contact the research team if applicable laws were overlooked. None of the jurisdictions indicated that laws were overlooked in the assessment.

Seven states (New Jersey, New York, North Carolina, Pennsylvania, Utah, Washington, and Wisconsin) have laws requiring animal contact exhibits to provide hand sanitation stations (Table). However, state laws vary regarding the types of exhibits to which the requirements apply. For example, North Carolina’s laws apply to all animal contact exhibits, including petting zoos, pony rides, and poultry handling exhibits. Wisconsin’s law, however, applies only to petting zoos located at campgrounds.

**TABLE:** Laws requiring hand sanitation stations at animal contact exhibits in seven states — United States, March–April 2016

*State*	*Citation*	*Applicable facilities*	*Handwashing station required*	Sign* recommending sanitation or indicating risk r*equired
*New Jersey*	N.J. Admin. Code Sect. 2:76-2A.13	Farm-based recreational activities at commercial farms	Yes*	Yes^†^
*New York*	N.Y. McKinney's Public Health Law Sect.1311; N.Y. McKinney's Public Health Law Sect. 12	Public establishments featuring animals	Yes	Yes
*New York*	N.Y. McKinney's General Business Law Sect. 399-ff	Petting zoos	Yes	Yes
*New York*	N.Y. Comp. Codes Rules and Regulations, Title 10, Sect. 7-5.1 - 5.15	Petting zoos at agricultural fairgrounds	Yes	Yes^§^
*North Carolina*	N.C. Gen. Stat. Ann. Sect. 106-520.1-72 N.C. Admin. Code 52K.0101-0702	Animal exhibitions at agricultural fairs	Yes	Yes
*Pennsylvania*	3 Pa. Code Sect. 2501-2504	Animal exhibitions	Yes	Yes
*Utah*	Utah Admin. Code R58-6; R58-19-4	Public exhibitions of poultry	Yes	Yes
*Washington*	Wash. Admin. Code Sect. 246-100-192; 246-100-070	Animal venue operators	Yes	Yes
*Wisconsin*	Wis. Admin. Code DHS Sect. 178.03, 178.18, 178.08, 178.07; Wis. Stat. Ann. Sect. 254.47	Petting zoos at campgrounds	Yes	Yes

* New Jersey’s law recommends and requires handwashing stations for commercial farms seeking to receive the protections of the New Jersey Right to Farm Act, NJSA 4:1C-1 et seq.

^†^ New Jersey’s law does not specifically mention signs but requires that visitors be advised to sanitize their hands, which is likely done via signage.

^§^ Per N.Y. McKinney’s General Business Law ^§^ 399-ff, which applies to petting zoos in the state, New York’s law requires recommending hand sanitation to patrons.

Laws in four of the seven states (New York, North Carolina, Pennsylvania, and Wisconsin) specify where the handwashing stations must be located in relation to the exhibit. These provisions vary as to the specific location. For example, North Carolina requires that a handwashing station be located within 10 feet (3 meters) of the exit of the exhibit when feasible, whereas Pennsylvania requires that the station be conveniently located on the animal exhibition grounds.

All seven states require that animal contact exhibits have signs recommending hand sanitation, or indicating the health risk for contact with animals. Four states (New York, North Carolina, Pennsylvania, and Wisconsin) require that signs indicating the location of the hand sanitation stations be placed at the exhibit.

The statutory or regulatory code in all seven states authorizes penalties against operators of animal contact exhibits for noncompliance with hand sanitation station laws. For example, in Pennsylvania, noncompliance is subject to a civil penalty of $500. In Wisconsin, campground petting zoo operators who are in violation are subject to suspension or revocation of their permits.

## Discussion

Law has played a demonstrable role in the great public health achievements of the 20th century, such as improvements in motor-vehicle safety and immunization, meriting research into its potential use in other areas of public health, including animal contact exhibit outbreaks (*6*,*7*). The results of this assessment highlight the depth and breadth of state laws related to hand sanitation stations at animal contact exhibits, including the type of exhibits, locations of the stations, signage requirements, and penalties. Within the seven jurisdictions that have these laws, the types of facilities covered by the laws vary. Some jurisdictions’ laws apply broadly to various facilities, whereas others apply only to a single facility type, such as petting zoos.

This study is subject to at least two limitations. First, although only seven states have established requirements for hand sanitation through statutes or regulations, states might be using other law or policy interventions not captured in this assessment to reduce the incidence of disease transmission at animal contact exhibits. For example, the assessment did not include a study of case law, administrative decisions, agency policies, or local laws. Second, this assessment did not study the implementation or enforcement of the statutory and regulatory requirements, which can influence the effectiveness of legal interventions. Despite these limitations, this assessment, a type of legal epidemiologic study, can increase the body of evidence-based research on the effectiveness of these legal interventions (*6*). Thus, the results of this assessment can be used by researchers in evaluating the public health impact of animal contact exhibit laws related to hand sanitation.

Proper handwashing is an effective way to prevent transmission of disease to persons at animal exhibits (*4*); however, outbreaks at animal contact exhibits continue to occur, in part because of a lack of handwashing stations. Statutory and regulatory interventions are tools that states use to address this preventable health risk. The results of this assessment of state laws related to hand sanitation at animal contact exhibits can be used as a tool for other jurisdictions interested in establishing similar laws.

**Summary**

**What is already known about this topic?**

Disease transmission linked to petting zoos, agricultural fairs, and other animal contact exhibits continues to be associated with outbreaks in the United States and can be minimized by proper handwashing after contact with animals. Some states have used law as a public health intervention to reduce the incidence of disease outbreaks associated with animal contact exhibits.

**What is added by this report?**

Seven states require hand sanitation stations for certain animal contact exhibits through statute or regulation. These statutes and regulations also require signs indicating location of the hand sanitation stations, or recommending hand sanitation, or provide penalties for violation of applicable laws.

**What are the implications for public health practice?**

This report can be used as a tool for states in establishing hand sanitation laws for animal contact exhibits in their own jurisdictions, and as data for researchers in evaluating the effectiveness of these laws.
